# Beyond the Skin: Atopic Dermatitis and Increased Gastric Cancer Risk in Korea

**DOI:** 10.3390/cancers17193214

**Published:** 2025-10-02

**Authors:** Ho Suk Kang, Kyeong Min Han, Joo-Hee Kim, Ji Hee Kim, Hyo Geun Choi, Dae Myoung Yoo, Ha Young Park, Nan Young Kim, Mi Jung Kwon

**Affiliations:** 1 Division of Gastroenterology, Department of Internal Medicine, Hallym University Sacred Heart Hospital, Hallym University College of Medicine, Anyang 14068, Republic of Korea; hskang76@hallym.or.kr; 2 Hallym Data Science Laboratory, Hallym University College of Medicine, Anyang 14068, Republic of Korea; km.han@hallym.ac.kr (K.M.H.); ydm@hallym.ac.kr (D.M.Y.); 3 Division of Pulmonary, Allergy, and Critical Care Medicine, Department of Internal Medicine, Hallym University Sacred Heart Hospital, Hallym University College of Medicine, Anyang 14068, Republic of Korea; luxjhee@hallym.or.kr; 4 Department of Neurosurgery, Hallym University Sacred Heart Hospital, Hallym University College of Medicine, Anyang 14068, Republic of Korea; kimjihee@hallym.or.kr; 5 Suseo Seoul E.N.T. Clinic, 10, Bamgogae-ro 1-gil, Gangnam-gu, Seoul 06349, Republic of Korea; mdanalytics@naver.com; 6 Department of Pathology, Busan Paik Hospital, Inje University College of Medicine, Busan 47392, Republic of Korea; mint@inje.ac.kr; 7 Hallym Institute of Translational Genomics and Bioinformatics, Hallym University Medical Center, Anyang 14068, Republic of Korea; honeyny@hallym.or.kr; 8 Department of Pathology, Hallym University Sacred Heart Hospital, Hallym University College of Medicine, Anyang 14068, Republic of Korea

**Keywords:** gastric cancer, atopic dermatitis, risk factor, nationwide population-based study, big data, Korea

## Abstract

**Simple Summary:**

Atopic dermatitis (AD) is a common inflammatory skin disease that has increasingly been recognized as a systemic disorder rather than a condition limited to the skin. While previous studies from Western countries have linked AD mainly to skin cancers and lymphomas, evidence regarding its relationship with gastrointestinal cancers, particularly gastric cancer (GC), is scarce. Using a large, nationwide Korean database, we found that AD was significantly associated with an increased risk of GC, especially among older adults, men, and rural residents. These results suggest that AD may serve as a novel risk factor for GC in East Asian populations with high disease burden. Our findings highlight the need for integrated dermatologic and gastroenterologic care, and they provide important insights for cancer prevention and risk stratification strategies.

**Abstract:**

Background/Objectives: Atopic dermatitis (AD) is a prevalent chronic inflammatory skin disease, but its relationship with gastric cancer (GC) remains unclear. This study aimed to investigate the association between AD and GC using a nationwide Korean database. Methods: Using the Korean National Health Insurance Service-National Sample Cohort, we conducted a nested case–control study including 10,174 GC patients and 40,696 matched controls (1:4 by age, sex, income, and region). Overlap propensity score weighting was used to control for confounders. Adjusted odds ratios (ORs) and 95% confidence intervals (CIs) were estimated via logistic regression. Results: AD was significantly associated with an increased risk of GC (adjusted OR = 1.08; 95% CI: 1.01–1.15). Subgroup analyses revealed stronger associations among individuals aged ≥ 65 years (OR = 1.12), men (OR = 1.10), rural residents (OR = 1.14), and those without comorbidities (CCI = 0, OR = 1.15). Higher risks were also observed in participants with non-allergic rhinitis (OR = 1.43) or no asthma (OR = 1.12). Conclusions: AD may be associated with an increased risk of GC in the Korean population. These findings may highlight the importance of considering dermatological conditions in the context of systemic cancer risk.

## 1. Introduction

Atopic dermatitis (AD) is one of the most common inflammatory skin diseases, affecting approximately 20% of children and 2–5% of adults worldwide, with particularly high prevalence reported in East Asian populations [1,2]. In South Korea, the prevalence among children aged 12 to 15 years increased from 7.2% in 1995 to 9.3% in 2000, and similar upward trends have been observed in Taiwan and China across different age groups [3]. These data highlight the growing public health burden of AD in the region. In moderate to severe cases, AD is associated not only with reduced sleep, impaired concentration, and diminished quality of life but also with a range of systemic comorbidities, including gastrointestinal disorders [4,5].

Beyond allergic comorbidities such as asthma and rhinitis, AD has been increasingly linked to chronic inflammation–related disorders, cardiovascular disease, and malignancy [6]. Large-scale epidemiological studies from Western countries provide mixed evidence. A UK population-based study reported a 49% higher overall risk of cancer in AD patients [7], while a Swedish register-based cohort also found elevated risks, particularly for lymphoma [8]. Similarly, Danish follow-up and nationwide studies showed increased risks of keratinocyte cancers and lymphomas, with stronger associations among patients with severe AD [9,10]. Two meta-analyses synthesizing these predominantly Western cohorts reported increased risks of squamous cell carcinoma of the skin and renal cancer but inconsistent associations for pancreatic and central nervous system cancers, limited by considerable heterogeneity across included studies [11,12]. Importantly, gastrointestinal cancers, including gastric cancer (GC), were not specifically evaluated in these analyses.

Evidence directly examining AD and GC is sparse. Swedish and Indian studies did not identify significant associations between AD and GC [8,13]. In East Asia, where GC incidence is among the highest worldwide, this is particularly relevant, as the region accounts for over 60% of global cases with markedly higher incidence and mortality than other regions [14]. GC is the fifth most prevalent cancer and the fourth leading cause of cancer-related deaths worldwide, accounting for 5.6% of all cancers [15,16]. South Korea, one of the countries with the highest incidence, has achieved a 21% reduction in GC mortality through nationwide screening programs [17]. Despite overall declines in incidence and mortality, a rising trend has been observed among individuals younger than 50 years [16,18].

Two Korean studies have provided preliminary but conflicting findings. A 2018 cross-sectional analysis suggested a reduced risk of GC among men with AD [19], whereas a 2023 nationwide cohort study found that allergic diseases overall were inversely associated with GC risk, but AD itself was not significantly linked [20]. Importantly, the latter study included AD as part of a broader allergic disease category and did not specifically examine its independent effect [20]. Both studies were limited by imbalanced sample sizes and demographic heterogeneity, which may have influenced the observed associations [21]. Given the high prevalence of GC in Korea alongside the increasing trend of AD [3,15], clarifying their relationship is clinically important.

Biologically, AD may influence gastric carcinogenesis through systemic immune dysregulation [22]. Asian AD phenotypes, in particular, demonstrate stronger Th17/Th22 activation compared with Western populations [23], potentially amplifying gastrointestinal mucosal inflammation [22]. AD is also closely linked to gastrointestinal health, with higher rates of comorbidities such as eosinophilic esophagitis, food allergy, inflammatory bowel disease, celiac disease, and irritable bowel syndrome [5]. Moreover, gut microbiota studies reveal reduced microbial diversity and enrichment of pro-inflammatory taxa such as *Staphylococcus aureus* and *Clostridium* in AD patients, alongside depletion of protective species such as *Bifidobacterium* [24]. These alterations may disrupt immune tolerance, sustain systemic inflammation, and plausibly increase susceptibility to GC.

Taken together, these epidemiological inconsistencies and mechanistic clues underscore the need to clarify whether AD confers an increased risk of GC in East Asian populations, where GC incidence remains high [14]. However, prior studies have either focused primarily on Western populations [8] or assessed allergic diseases as a whole, without specifically isolating the independent effect of AD on GC [20]. This lack of direct evidence in East Asia represents a significant scientific gap. Therefore, our study aimed to fill this gap by investigating the association between AD and GC using a nationwide, population-based case–control design with 1:4 propensity score matching, thereby providing robust and balanced evidence.

## 2. Materials and Methods

### 2.1. Ethics, Data Source, and Study Design

The study protocol was approved by the Institutional Review Board of Hallym University Sacred Heart Hospital (IRB No. 2022-10-008), and the requirement for written informed consent was waived due to the use of de-identified data. All study procedures were conducted in accordance with relevant ethical guidelines and regulations. To investigate the relation between the history of subjects and the presence or absence of exposure in outcome status, a nested case–control study design was deemed appropriate [25].

This retrospective nested case–control study was conducted using the Korean National Health Insurance Service–National Sample Cohort (KNHIS-NSC), a representative dataset comprising approximately 2.2% of the Korean population followed from 2002 to 2019 [26]. The cohort was constructed by random sampling and stratified into 1476 strata according to age (18 groups), sex, and income level (41 groups), ensuring national representativeness. Participants were followed until 2019 unless censored by death or emigration. The KNHIS-NSC provides a large, stable, and comprehensive dataset generated from nationwide insurance claims and government health records.

### 2.2. Definition of Gastric Cancer (Outcome)

GC was defined as the concurrent presence of an ICD-10 diagnostic code for GC (C16) and national cancer-specific billing codes (V193 or V194). These V-codes are assigned only to patients with confirmed cancer diagnoses registered in the national insurance system to receive financial support for critical illnesses. This dual-coding strategy, widely applied in Korean administrative database research, enhances diagnostic specificity and minimizes misclassification [27].

### 2.3. Definition of Atopic Dermatitis (Exposure)

AD was defined as a diagnosis of AD recorded under ICD-10 code L20.

### 2.4. Participant Selection and Matching

**Figure 1:**
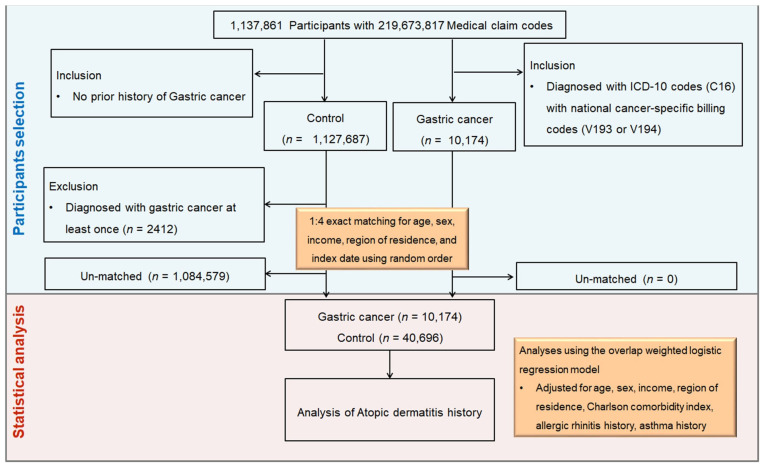
Schematic illustration of the participant selection process in the present study. Among a total of 1,137,861 participants, 10,174 gastric cancer participants were finally matched with 40,696 control participants for age, sex, income, and region of residence.

Between 2002 and 2019, a total of 1,137,861 participants and 219,673,817 medical claim records were included in the KNHIS-NSC. From these, we identified 10,174 patients with newly diagnosed GC between 2005 and 2019. Individuals not diagnosed with gastric cancer from 2005 to 2019 were included in the control group (*n* = 1,127,687). After excluding 2412 potentially misclassified individuals, we performed 1:4 exact matching based on age, sex, income level, and residential area, excluding 1,084,579 un-matched controls. The index date for each GC case, defined as the date of both C16 and V-code assignment, was assigned to the corresponding matched controls. In total, 50,870 participants (10,174 GC cases and 40,696 controls) were included in the analysis (Figure 1). To further adjust for residual confounding, we estimated propensity scores using baseline covariates and applied overlap weighting to achieve covariate balance.

### 2.5. Covariates

Baseline characteristics included age (18 groups, 5-year intervals), sex, income level (five categories), and residential area (16 administrative districts, further classified as urban or rural). Comorbidity burden was assessed using the Charlson Comorbidity Index (CCI), calculated from 17 predefined conditions with scores ranging from 0 to 29, excluding cancer to prevent overlap with the outcome [28]. Allergic rhinitis (ICD-10: J30.1–J30.4) and asthma (ICD-10: J45–J46) were also identified as covariates.

### 2.6. Statistical Analyses

To balance baseline characteristics between the GC and control groups and to improve the precision of estimates, propensity score overlap weighting was applied. Propensity scores were estimated using multivariable logistic regression including all baseline covariates. Overlap weights were assigned as 1—propensity score for individuals in the GC group and as propensity score for those in the control group [29]. This approach ensured covariate balance while maximizing the effective sample size. Covariate balance between groups was assessed using standardized differences, with an absolute standardized difference <0.20 considered indicative of satisfactory balance [30]. To evaluate the association between GC and AD, a multivariable logistic regression analysis with overlap weighting was conducted to estimate odds ratios (ORs) and 95% confidence intervals (CIs). Both a crude model (unadjusted) and an adjusted model (adjusted for age, sex, income level, region of residence, and CCI score) were analyzed.

The association between AD and GC was evaluated using multivariable logistic regression with overlap weighting to estimate odds ratios (ORs) and 95% confidence intervals (CIs). Both crude (unadjusted) and adjusted models (adjusted for age, sex, income level, region of residence, and CCI score) were analyzed. Subgroup analyses were further performed across all covariates to examine consistency of associations. All analyses were conducted using SAS software, version 9.4 (SAS Institute, Cary, NC, USA). Two-sided *p*-values < 0.05 were considered statistically significant.

## 3. Results

### 3.1. Baseline Characteristics

Table 1 presents the baseline characteristics of 10,174 patients with GC and 40,696 matched controls, matched at a 1:4 ratio on the basis of age, sex, income level, and region of residence. After matching, all covariates showed a standardized difference of 0.00, indicating a high degree of balance between groups. Additional overlap weighting further reduced standardized differences for all included covariates to 0, reflecting excellent covariate balance across the GC and control groups. Key variables such as the CCI, allergic rhinitis, and asthma were also well balanced. These results suggest that the matching and weighting procedures were effective in minimizing confounding factors and enhancing the validity of the subsequent analyses.

### 3.2. Relationship Between Gastric Cancer and Atopic Dermatitis

As shown in Table 2, to evaluate the associations between GC and AD, a propensity score overlap-weighted multivariable logistic regression analysis was conducted. With a full adjustment of confounding factors, AD was significantly associated with an increased risk of GC (adjusted OR = 1.08; 95% CI = 1.01–1.15; *p* = 0.027).

### 3.3. Subgroup Analyses

**Figure 2:**
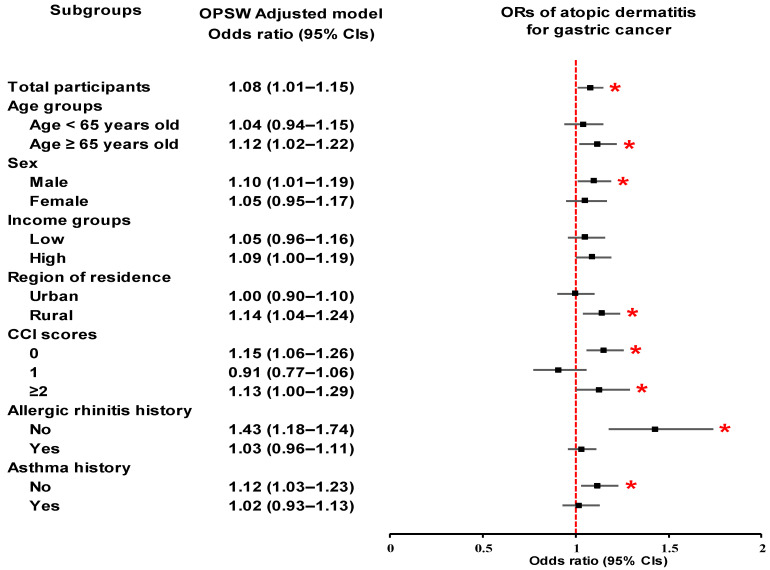
Forest plot of odds ratios (95% confidence intervals) for the association between atopic dermatitis and gastric cancer, including subgroup analyses by age, sex, income, residential area, Charlson comorbidity index, allergic rhinitis history, and asthma history. * Significance at *p* < 0.05.

Subgroup analyses consistently demonstrated a statistically significant association between AD and an increased risk of GC in specific demographic and clinical subgroups (Table 2; Figure 2). Significant associations were observed among participants aged ≥ 65 years (OR, 1.12; 95% CI, 1.02–1.22; *p* = 0.015), males (OR, 1.10; 95% CI, 1.01–1.19; *p* = 0.028), and those residing in rural areas (OR, 1.14; 95% CI, 1.04–1.24; *p* = 0.003). Among individuals with no comorbidities (CCI = 0), the association was also statistically significant (OR, 1.15; 95% CI, 1.06–1.26; *p* = 0.001). Furthermore, participants with a history of non-allergic rhinitis (OR, 1.43; 95% CI, 1.18–1.74; *p* < 0.001) or no history of asthma (OR, 1.12; 95% CI, 1.03–1.23; *p* = 0.011) presented elevated risk.

## 4. Discussion

In this large nationwide case–control study, we found that AD was significantly associated with an increased risk of GC after rigorous adjustment for confounders using overlap weighting. The association was particularly evident among men, older adults, rural residents, and individuals with fewer comorbidities. These findings add important evidence to the limited and inconsistent literature on the relationship between AD and GC, highlighting AD as a potential novel risk factor in East Asian populations, where GC incidence remains among the highest worldwide.

Our findings both expand upon and diverge from previous studies. Several large-scale Western cohorts, including a U.K. study using the THIN database and a Swedish register-based analysis, reported elevated risks of lymphoma, melanoma, and keratinocyte cancers in AD patients, but did not demonstrate consistent associations with gastrointestinal malignancies, including GC [7,8]. Similarly, a Mendelian randomization study in European populations found no causal relationship between AD and digestive cancers, although GC was not specifically assessed [31]. These discrepancies underscore the complexity of AD–cancer associations and the importance of region-specific investigations.

In Asia, the evidence has been heterogeneous. A Taiwanese cohort showed that the cancer risk associated with allergic diseases, including AD, varied depending on cancer type, but no clear link with GC was observed [32]. An Indian cross-sectional study also reported a nonsignificant inverse association between AD and GC in men [13]. In Korea, a 2018 cross-sectional analysis suggested a reduced but nonsignificant risk of GC among men with AD [19], whereas a 2023 population-based cohort found that allergic diseases collectively—including AD—were inversely associated with GC, but AD alone was not significantly linked [20]. These discrepancies may be attributable to methodological limitations, including cross-sectional designs, heterogeneous definitions of allergic disease, smaller sample sizes, and inadequate confounder control [7,8,19,20,32]. In contrast, our study leveraged a large, nationally representative Korean cohort, used strict case definitions with dual diagnostic and billing codes, and applied robust propensity score matching with overlap weighting, thereby enhancing diagnostic accuracy, temporal validity, and covariate balance.

Significant associations were observed among participants aged ≥65 years (OR, 1.12; 95% CI, 1.02–1.22; *p* = 0.015), males (OR, 1.10; 95% CI, 1.01–1.19; *p* = 0.028), and those residing in rural areas (OR, 1.14; 95% CI, 1.04–1.24; *p* = 0.003). The elevated risk observed in rural populations may be attributable to several factors. Rural residents in Korea often have limited access to specialized dermatologic and gastroenterologic care, which can delay diagnosis and management of both AD and GC [33]. In addition, lower participation in national cancer screening programs among rural populations may further exacerbate this risk [17]. These findings suggest that rural populations with AD warrant particular attention in future cancer prevention and risk stratification strategies.

Interestingly, our subgroup analyses revealed that individuals with non-allergic rhinitis or without a history of asthma exhibited higher risks of GC. This finding may reflect distinct immunological mechanisms. Non-allergic rhinitis is often associated with chronic nasal inflammation mediated by non-IgE pathways, which may indicate broader systemic inflammatory activity contributing to gastric carcinogenesis. In contrast, asthma—particularly allergic asthma—has been hypothesized to confer some protective effects against certain cancers through heightened immune surveillance and increased Th2-mediated responses. The absence of asthma might therefore reflect reduced immunological activation, potentially explaining the elevated GC risk in this subgroup. Alternatively, these results may be influenced by heterogeneity in allergic disease phenotypes or differences in healthcare utilization patterns.

Several biological pathways may explain the observed association between AD and GC. First, systemic immune dysregulation in AD could play a central role. Asian AD phenotypes are characterized by stronger Th17/Th22-driven inflammation compared with Western populations [23,34]. IL-17 and IL-22, cytokines highly expressed in AD lesions, are also enriched in GC tissues [22,35,36] and are known to promote epithelial proliferation, angiogenesis, and NF-κB signaling [37,38], thereby creating a protumorigenic microenvironment. Second, AD is closely linked to gastrointestinal comorbidities such as eosinophilic esophagitis, food allergy, inflammatory bowel disease, celiac disease, and gastroesophageal reflux [5]. These conditions may contribute to chronic mucosal inflammation and altered immune tolerance, increasing susceptibility to gastric carcinogenesis.

Third, microbiome dysbiosis may represent an important mediator. Gut microbiota studies in AD patients have reported reduced bacterial diversity, depletion of protective taxa such as *Bifidobacterium*, and enrichment of pro-inflammatory species including *Escherichia coli* and *Clostridium* [24]. Such alterations may impair immune homeostasis, exacerbate systemic inflammation, and influence carcinogen metabolism.

From a clinical perspective, our findings suggest that AD should be regarded as more than a cutaneous disorder, with implications for systemic health and cancer risk. The particularly strong associations observed among older adults, men, rural residents, and those without comorbidities underscore the importance of targeted risk stratification in these subgroups. In South Korea, where nationwide endoscopic screening programs have already reduced GC mortality by 21% [17], prioritizing AD patients—particularly those belonging to high-risk subgroups identified in our analysis—could further improve screening efficiency. Beyond clinical care, these findings also have important public health implications. Recognizing AD as a potential risk factor for GC may inform cancer prevention campaigns and screening guidelines. Tailored public health initiatives for high-risk groups, coupled with the integration of dermatologic and gastroenterologic perspectives in health education and early detection programs, could ultimately contribute to reducing the burden of GC at the population level.

This study has several notable strengths. First, it utilized a large, nationally representative dataset with nearly 20 years of follow-up, providing robust statistical power and temporal validity. Second, the dual-coding strategy for GC ensured high diagnostic specificity, minimizing misclassification. Third, the application of 1:4 propensity score matching and overlap weighting allowed for rigorous adjustment of confounding factors, with excellent covariate balance achieved between groups. Finally, detailed subgroup analyses provided insight into population-level heterogeneity in risk.

Several limitations should be acknowledged. First, the use of administrative claims data may introduce misclassification, although our dual-code strategy mitigates this risk. Second, residual confounding by unmeasured factors such as family history, smoking, alcohol consumption, diet, and *H. pylori* infection cannot be entirely excluded. Third, disease severity of AD could not be stratified, and our findings may not be generalizable beyond the Korean population. Moreover, laboratory data on type 2 inflammatory markers, such as serum IgE or thymus and activation-regulated chemokine (TARC), were unavailable in our dataset. These biomarkers are closely linked to AD pathophysiology and could provide mechanistic insight into the observed association with GC. Future studies integrating clinical, genetic, microbiome, and biomarker data will be essential to validate and refine these associations and to further elucidate the role of type 2 inflammation in gastric carcinogenesis.

## 5. Conclusions

In summary, this nationwide case–control study provides evidence that AD is significantly associated with an increased risk of GC in the Korean population. By leveraging robust methodological approaches, our study demonstrates that AD may represent a novel, independent risk factor for GC, particularly in East Asian populations where GC burden is high. Recognition of AD as part of a systemic inflammatory spectrum may inform risk stratification, early detection strategies, and the development of preventive interventions at both the individual and population levels.

## Figures and Tables

**Table 1:** General Characteristics of Participants.

Characteristics		Before Overlap PS Weighting Adjustment			After Overlap PS Weighting Adjustment		
		GC	Control	Standardized Difference	GC	Control	Standardized Difference
Age (n, %)				0.00			0.00
	0–4	N/A	N/A		N/A	N/A	
	5–9	1 (0.01)	4 (0.01)		1 (0.01)	1 (0.01)	
	10–14	3 (0.03)	12 (0.03)		2 (0.02)	2 (0.02)	
	15–19	N/A	N/A		N/A	N/A	
	20–24	1 (0.01)	4 (0.01)		1 (0.01)	1 (0.01)	
	25–29	19 (0.19)	76 (0.19)		13 (0.17)	13 (0.17)	
	30–34	95 (0.93)	380 (0.93)		64 (0.86)	64 (0.86)	
	35–39	205 (2.01)	820 (2.01)		143 (1.91)	143 (1.91)	
	40–44	466 (4.58)	1864 (4.58)		340 (4.54)	340 (4.54)	
	45–49	711 (6.99)	2844 (6.99)		515 (6.88)	515 (6.88)	
	50–54	994 (9.77)	3976 (9.77)		716 (9.57)	716 (9.57)	
	55–59	1197 (11.77)	4788 (11.77)		872 (11.65)	872 (11.65)	
	60–64	1449 (14.24)	5796 (14.24)		1065 (14.23)	1065 (14.23)	
	65–69	1463 (14.38)	5852 (14.38)		1081 (14.45)	1081 (14.45)	
	70–74	1490 (14.65)	5960 (14.65)		1110 (14.84)	1110 (14.84)	
	75–79	1071 (10.53)	4284 (10.53)		802 (10.72)	802 (10.72)	
	80–84	693 (6.81)	2772 (6.81)		519 (6.94)	519 (6.94)	
	85+	316 (3.11)	1264 (3.11)		239 (3.19)	239 (3.19)	
Sex (n, %)				0.00			0.00
	Male	6834 (67.17)	27,336 (67.17)		5032 (67.26)	5032 (67.26)	
	Female	3340 (32.83)	13,360 (32.83)		2449 (32.74)	2449 (32.74)	
Income (n, %)				0.00			0.00
	1 (lowest)	1959 (19.25)	7836 (19.25)		1429 (19.10)	1429 (19.10)	
	2	1260 (12.38)	5040 (12.38)		918 (12.27)	918 (12.27)	
	3	1621 (15.93)	6484 (15.93)		1190 (15.91)	1190 (15.91)	
	4	2144 (21.07)	8576 (21.07)		1567 (20.95)	1567 (20.95)	
	5 (highest)	3190 (31.35)	12,760 (31.35)		2376 (31.76)	2376 (31.76)	
Region of residence (n, %)				0.00			0.00
	Urban	4310 (42.36)	17,240 (42.36)		3168 (42.35)	3168 (42.35)	
	Rural	5864 (57.64)	23,456 (57.64)		4313 (57.65)	4313 (57.65)	
CCI score (Mean, SD)		2.40 (2.70)	1.02 (1.70)	0.61	1.82 (1.98)	1.82 (1.02)	0.00
Allergic rhinitis history (n, %)		796 (7.82)	3178 (7.81)	0.1	595 (7.95)	570 (7.62)	0.00
Asthma history (n, %)		7753 (76.20)	32,615 (80.14)	0.09	5836 (78.21)	5836 (78.21)	0.00
Atopic dermatitis (n, %)		3687 (36.24)	16,482 (40.5)	0.00	2821 (37.80)	2821 (37.80)	0.02

Abbreviations: CCI, Charlson Comorbidity Index; PS, propensity score; N/A, not applicable; SD, standard deviation.

**Table 2:** Crude and overlap propensity score-weighted odds ratios of atopic dermatitis for gastric cancer.

Characteristics		N of GC	N of Control	OR for GC (95% CI)			
		(AD Cases/Total, %)	(AD Cases/Total, %)	Crude	*p*	Overlap Weighted Model †	*p*
Total participants (n = 50,870)							
	AD	796/10,174 (7.8)	3178/40,696 (7.8)	1.00 (0.92–1.09)	0.96	1.08 (1.01–1.15)	0.027 *
	Control	9378/10,174 (92.2)	37,518/40,696 (92.2)	1		1	
Age < 65 years old (n = 25,705)							
	AD	340/5141 (6.6)	1435/20,564 (7.0)	0.94 (0.84–1.07)	0.356	1.04 (0.94–1.15)	0.441
	Control	4801/5141 (93.4)	19,129/20,564 (93.0)	1		1	
Age ≥ 65 years old (n = 25,165)							
	AD	456/5033 (9.1)	1743/20,132 (8.7)	1.05 (0.94–1.17)	0.366	1.12 (1.02–1.22)	0.015 *
	Control	4577/5033 (90.9)	18,389/20,132 (91.3)	1		1	
Male (n = 34,170)							
	AD	509/6834 (7.4)	1996/27,336 (7.3)	1.02 (0.92–1.13)	0.676	1.10 (1.01–1.19)	0.028 *
	Control	6325/6834 (92.6)	25,340/27,336 (92.7)	1		1	
Female (n = 16,700)							
	AD	287/3340 (8.6)	1182/13,360 (8.8)	0.97 (0.85–1.11)	0.645	1.05 (0.95–1.17)	0.347
	Control	3053/3340 (91.4)	12,178/13,360 (91.2)	1		1	
Low income group (n = 24,200)							
	AD	362/4840 (7.5)	1445/19,360 (7.5)	1.00 (0.89–1.13)	0.971	1.05 (0.96–1.16)	0.279
	Control	4478/4840 (92.5)	17,915/19,360 (92.5)	1		1	
High income group (n = 26,670)							
	AD	434/5334 (8.1)	1733/21,336 (8.1)	1.00 (0.90–1.12)	0.973	1.09 (1.00–1.19)	0.055
	Control	4900/5334 (91.9)	19,603/21,336 (91.9)	1		1	
Urban resident (n = 21,550)							
	AD	333/4310 (7.7)	1437/17,240 (8.3)	0.92 (0.81–1.04)	0.193	1.00 (0.90–1.10)	0.974
	Control	3977/4310 (92.3)	15,803/17,240 (91.7)	1		1	
Rural resident (n = 29,320)							
	AD	463/5864 (7.9)	1741/23,456 (7.4)	1.07 (0.96–1.19)	0.219	1.14 (1.04–1.24)	0.003 *
	Control	5401/5864 (92.1)	21,715/23,456 (92.6)	1		1	
CCI scores = 0 (n = 27,284)							
	AD	307/3480 (8.8)	1849/23,804 (7.8)	1.15 (1.01–1.30)	0.031 *	1.15 (1.06–1.26)	0.001 *
	Control	3173/3480 (91.2)	21,955/23,804 (92.2)	1		1	
CCI scores = 1 (n = 8937)							
	AD	142/1990 (7.1)	577/6947 (8.3)	0.85 (0.70–1.03)	0.091	0.91 (0.77–1.06)	0.222
	Control	1848/1990 (92.9)	6370/6947 (91.7)	1		1	
CCI scores ≥ 2 (n = 14,649)							
	AD	347/4704 (7.4)	752/9945 (7.6)	0.97 (0.85–1.11)	0.693	1.13 (1.00–1.29)	0.049 *
	Control	4357/4704 (92.6)	9193/9945 (92.4)	1		1	
Non-allergic rhinitis history (n = 10,502)							
	AD	114/2421 (4.7)	293/8081 (3.6)	1.31 (1.05–1.64)	0.016 *	1.43 (1.18–1.74)	<0.001 *
	Control	2307/2421 (95.3)	7788/8081 (96.4)	1		1	
Allergic rhinitis history (n = 40,368)							
	AD	682/7753 (8.8)	2885/32,615 (8.8)	0.99 (0.91–1.08)	0.891	1.03 (0.96–1.11)	0.354
	Control	7071/7753 (91.2)	29,730/32,615 (91.2)	1		1	
Non-asthma history (n = 30,701)							
	AD	442/6487 (6.8)	1568/24,214 (6.5)	1.06 (0.95–1.18)	0.328	1.12 (1.03–1.23)	0.011 *
	Control	6045/6487 (93.2)	22,646/24,214 (93.5)	1		1	
Asthma history (n = 20,169)							
	AD	354/3687 (9.6)	1610/16,482 (9.8)	0.98 (0.87–1.11)	0.758	1.02 (0.93–1.13)	0.63
	Control	3333/3687 (90.4)	14,872/16,482 (90.2)	1		1	

Abbreviations: GC, gastric cancer; AD, atopic dermatitis; OR, odds ratio; CI, confidence interval; CCI, Charlson Comorbidity Index; * Significance at *p* < 0.05. † Adjusted for age, sex, income, region of residence, CCI score, allergic rhinitis history, and asthma history.

## Data Availability

Restrictions apply to the availability of these data. The data were obtained from the Korean National Health Insurance Sharing Service (NHISS) and are available at https://nhiss.nhis.or.kr (accessed in 1 May 2025).
